# Considerations and code for partial volume correcting [^18^F]-AV-1451 tau PET data

**DOI:** 10.1016/j.dib.2017.10.024

**Published:** 2017-10-16

**Authors:** Suzanne L. Baker, Anne Maass, William J. Jagust

**Affiliations:** a Molecular Biophysics and Integrated Bioimaging, Lawrence Berkeley National Laboratory, Berkeley, CA, United States; b Helen Wills Neuroscience Institute, University of California, Berkeley, Berkeley, CA, United States; c German Center for Neurodegenerative Diseases, Magdeburg, Germany

## Abstract

[^18^F]-AV-1451 is a leading tracer used with positron emission tomography (PET) to quantify tau pathology. However, [^18^F]-AV-1451 shows “off target” or non-specific binding, which we define as binding of the tracer in unexpected areas unlikely to harbor aggregated tau based on autopsy literature [1]. Along with caudate, putamen, pallidum and thalamus non-specific binding [2], [3], we have found binding in the superior portion of the cerebellar gray matter, leading us to use inferior cerebellar gray as the reference region. We also addressed binding in the posterior portion of the choroid plexus. PET signal unlikely to be associated with tau also occurs in skull, meninges and soft tissue (see e.g. [4]). We refer to [^18^F]-AV-1451 binding in the skull and meninges as extra-cortical hotspots (ECH) and find them near lateral and medial orbitofrontal, lateral occipital, inferior and middle temporal, superior and inferior parietal, and inferior cerebellar gray matter. Lastly, the choroid plexus also shows non-specific binding that bleeds into hippocampus. We are providing the code (http://www.runmycode.org/companion/view/2798) used to create different regions of interest (ROIs) that we then used to perform Partial Volume Correction (PVC) using the Rousset geometric transfer matrix method (GTM, [5]). This method was used in the companion article, “Comparison of multiple tau-PET measures as biomarkers in aging and Alzheimer's Disease” ([6], DOI 10.1016/j.neuroimage.2017.05.058).

**Specifications Table**

**Table:** 

Subject area	*Analysis Methods*
More specific subject area	*[*^*18*^*F]-AV-1451 imaging*
Type of data	*Table, Matlab code*
How data was acquired	*PET*
Data format	*Matlab code*
Experimental factors	*n/a*
Experimental features	*Subjects were scanned 80–100 minutes after injection of [18 F]-AV-1451. 4 × 5 min frames were reconstructed, realigned, summed and coregistered to an MRI. FreeSurfer, SUIT cerebellar template, and SPM12 segmentation was used to create regions of interest (ROIs) used to partial volume correct the data using the Rousset method.*
Data source location	http://www.runmycode.org/companion/view/2798
Data accessibility	*Code runs in Matlab*
Related research article	*Companion article: Comparison of multiple tau-PET measures as biomarkers in aging and Alzheimer's Disease*
	*PMID 28587897*
	*DOI*10.1016/j.neuroimage.2017.05.058

**Value of the data**

– [^18^F]-AV1451, although useful, displays some nuisance off-target binding that, due to partial volume effects, affects regions of the brain (temporal cortex, hippocampus, reference region) that are of great interest in studying tau as it relates to aging and disease.
– Fundamentally we are providing the code (uploaded with the article) that will create an ideal set of ROIs for studying tau deposition, along with a set of ROIs that must be accounted for if proper partial volume correction is to be performed.
– This code addresses the reference region in two ways: removing superior portion of the cerebellar gray which typically shows [^18^F]-AV-1451 binding thereby contaminating the reference region, and also scans the remaining reference region (inferior cerebellar gray) for the rare occasion of isolated increased [^18^F]-AV-1451 binding.
– The PVC code specifically addresses uptake in the choroid plexus, splitting choroid plexus into 2 clusters of higher and lower uptake. It also addresses off-target binding of tracer in the skull creating clusters of extra cortical hotspots (ECH) used as ROIs in the partial volume correction algorithm.

## 1 Data

We are sharing the code (http://www.runmycode.org/companion/view/2798) used to create regions of interest and deploy the GTM approach for partial volume correction [5] of [^18^F]-AV-1451 tau PET data. This code creates an inferior cerebellar gray reference region, splits the choroid plexus region into high and low binding, creates subject-specific extra-cortical hotspot regions, creates high and low binding CSF and skull+meninges regions.

## 2 Experimental design, materials and methods

10 young and middle-aged healthy subjects (YC), 83 older healthy controls (OC) and 68 subjects with MCI or AD (MCI/AD) were used in this analysis (Table 1). Subjects were a subset of Sample 1 subjects used in [6]. MRI and [^18^F]-AV-1451 tau PET scans were acquired at Lawrence Berkeley National Laboratory [7]. MRIs were segmented using FreeSurfer version 5.3 (http://surfer.nmr.mgh.harvard.edu/) resulting in ROI segmentation (aparc+aseg.nii) file. We further segmented the cerebellum by using SPM12 (www.fil.ion.ucl.ac.uk/spm) to reverse normalize the SUIT cerebellar template (http://www.diedrichsenlab.org/imaging/suit.htm) to each subject's native space. MRIs were also segmented into gray (c1), white (c2), cerebrospinal fluid (c3), skull and meninges (c4 and c5) probability maps using SPM12. The mean [^18^F]-AV-1451 data were coregistered to the MRI.

**Table 1:** Subjects.

	Gender	Mean Age and range
Young Healthy Controls (YC)	9 Males / 1 Female	36.6 (20.5–56.5)
Older Healthy Controls (OC)	33 Males / 50 Females	77.1 (60–94.8)
MCI and AD (MCI/AD)	30 Males / 38 Females	65.1 (47.7–83.4)

The purpose of the code is to thoughtfully create 1. a set of ROIs that explore interesting scientific questions, and 2. a set of ROIs where nuisance off-target binding occurs resulting in partial volume effects in the former ROIs. We will focus more on 2. In order to test the effect of specific steps in the ROI creation for the PVC, we tested 10 different ROI configurations:

1. FreeSurfer ROIs grouped as shown in Table 2 plus the whole choroid plexus. Any voxel not defined by FreeSurfer (cerebrospinal fluid (CSF), skull, meninges) was assumed to be 0.

**Table 2:** index in edited_aparc+aseg created by program, corresponding FreeSurfer index and FreeSurfer name of ROI. Braak ROI mean values [6] were calculated across the following indexes: Braak I/II: Voxel index 1:4; Braak III/IV: Voxel index 5:30 (excluding 14, 23), Braak V/VI: Voxel index 31:72 (excluding 35,36,41,45,51,52,57,61).

Voxel index	FreeSurfer index	Region	Voxel index	FreeSurfer index	Region
**1**	1006	L Entorhinal Cortex	**40**	1030	L Superior Temporal
**2**	2006	R Entorhinal Cortex	**41**	13	L Pallidum
**3**	17	L Hippocampus	**42**	1029	L Parietal Superior
**4**	53	R Hippocampus	**43**	1025	L Precuneus
**5**	1016	L Parahippocampal	**44**	1001	L BankSTS
**6**	1007	L Fusiform	**45**	26	L Accumbens
**7**	1013	L Lingual	**46**	1034	L Tranv Temporal
**8**	18	L Amygdala	**47**	2028	R Frontal SUPFR
**9**	2016	R Parahippcampal	**48**	2012, 2014, 2032	R Frontal FPORB
**10**	2007	R Fusiform	**49**	2003, 2027	R Frontal MIDFR
**11**	2013	R Lingual	**50**	2018, 2019, 2020	R Frontal PARSFR
**12**	54	R Amygdala	**51**	50	R Caudate
**13**	1015	L Middle Temporal	**52**	51	R Putamen
**14**	10	L Thalamus	**53**	2011	R Lateral Occipital
**15**	1002	L CaudAnt Cingulate	**54**	2031	R Parietal Supramarginal
**16**	1026	L RostAnt Cingulate	**55**	2008	R Parietal Inferior
**17**	1023	L Post Cingulate	**56**	2030	R Superior Temporal
**18**	1010	L Isthmus Cingulate	**57**	52	R Pallidum
**19**	1035	L Insula	**58**	2029	R Parietal Superior
**20**	1009	L Inferior Temporal	**59**	2025	R Precuneus
**21**	1033	L Temporal Pole	**60**	2001	R bankSTS
**22**	2015	R Middle Temporal	**61**	58	R Accumbens
**23**	49	R Thalamus	**62**	2034	R Tranv Temporal
**24**	2002	R CaudAnt Cingulate	**63**	1021	L Pericalcarine
**25**	2026	R RostAnt Cingulate	**64**	1022	L Postcentral
**26**	2023	R Post Cingulate	**65**	1005	L Cuneus
**27**	2010	R Isthmus Cingulate	**66**	1024	L Precentral
**28**	2035	R Insula	**67**	1017	L Paracentral
**29**	2009	R Inferior Temporal	**68**	2021	R Pericalcarine
**30**	2033	R Temporal Pole	**69**	2022	R Postcentral
**31**	1028	L Frontal SUPFR	**70**	2005	R Cuneus
**32**	1012, 1014, 1032	L Frontal FPORB	**71**	2024	R Precentral
**33**	1003, 1027	L Frontal MIDFR	**72**	2017	R Paracentral
**34**	1018, 1019, 1020	L Frontal PARSFR	**73**	2, 41, 251–255	Hemispherical White
**35**	11	L Caudate	**74**	7, 46	Cerebellar White
**36**	12	L Putamen	**75**	16	Brainstem
**37**	1011	L Lateral Occipital	**76**	28, 30, 60, 62, 77, 80, 85, 1000, 1004, 2000, 2004	Other
**38**	1031	L Parietal Supramarginal	**77**	Some of 8,47	Superior Cerebellar Gray
**39**	1008	L Parietal Inferior	**0**	4,5,14,15,24,43,44,72	Ventricles
2. Same as 1, but choroid plexus was split into high and low choroid plexus ROIs. We found choroid plexus uptake was higher in the ventral portion that was next to the hippocampus, and lower in the dorsal portion and wanted to explore the effect of segmenting this ROI into high and low on the PVC.
3. Same as 2, but also included extra-cortical hotspots (ECH). ECHs were determined on a subject-by-subject basis. An ECH was defined as a cluster of >500 voxels with SUVR>1.6, each ECH was added as its own ROI for PVC.
4. Same as 2, but included an ROI for SPM12 c3 (CSF) and another ROI for c4+c5 (skull + meninges), no ECHs.
5. Same as 3, ECHs with threshold of 1.6, but included an ROI for SPM12 c3 and another ROI for c4+c5 (as described in step 4).
6. Same as 4, but the c3 mask was divided into a c3 high (SUVR≥1) and c3 low (SUVR<1), and c4+c5 mask was divided into c4+c5 high (SUVR≥1) and c4+c5 low (SUVR<1) masks. Did not include ECHs.
7. Same as 5, but the c3 mask was divided into a c3 high (SUVR≥1) and c3 low (SUVR<1), and the c4+c5 mask was divided into c4+c5 high (SUVR≥1) and c4+c5 low (SUVR<1) masks. Included ECHs using 1.6 as threshold.
8. Same as 7 with ECH threshold was 1.6, and added a search to remove voxels > 1.6 from inferior cerebellar gray.
9. Same as 7, but ECH threshold was 1.3 and added a search to remove voxels > 1.6 from inferior cerebellar gray.
10. Same as 7, but ECH threshold was 1.9 and added a search to remove voxels > 1.6 from inferior cerebellar gray.

**Fig. 1:**
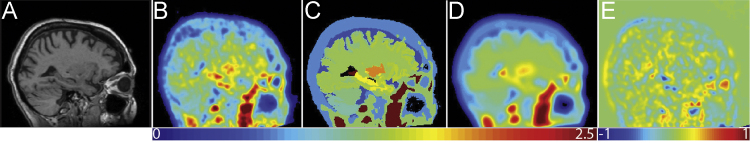
OC subject. A: MPRage. B: Corresponding [18 F]-AV-1451 scan with no PVC. C: Rousset PVC image using ROI group 7. D: Calculated pre-PVC, C smoothed to the resolution of the scanner. E: Calculated pre-PVC – original SUVR (D-B).

In order to quantify the success of PVC with the 10 different ROI configurations, within a subject and ROI configuration, a PVC image was created (example: Fig. 1C), in which the PV-corrected SUVR value of each ROI was assigned to all voxels within that ROI. This image was then smoothed by the resolution of the scanner to create a calculated pre-PVC image (example: Fig. 1D). If the ROI configuration and smoothing kernel of the scanner explained the original SUVR perfectly, the calculated pre-PVC image would look exactly the same as the original SUVR, although estimation of the smoothing kernel, subject motion, inhomogeneity of ROIs, coregistration between PET and MRI, and imperfect segmentation of the MRI are a few possible sources of error. The difference between the original SUVR and the calculated pre-PVC image was calculated (example: Fig. 1E), these are the residuals. The mean within ROIs of the difference between original SUVR and calculated pre-PVC is 0, or close to 0, and does not offer any information as to how well the PVC ROI configuration fits the original data. However, the standard deviation within an ROI is useful in quantifying the performance of an PVC ROI configuration; it is lower when the PVC configuration fits the data better and higher for worse fits. We normalized the within ROI standard deviation by the mean of the original SUVR (PVCnstd) in order to quantify how well an ROI configuration is performing.

**Fig. 2:**
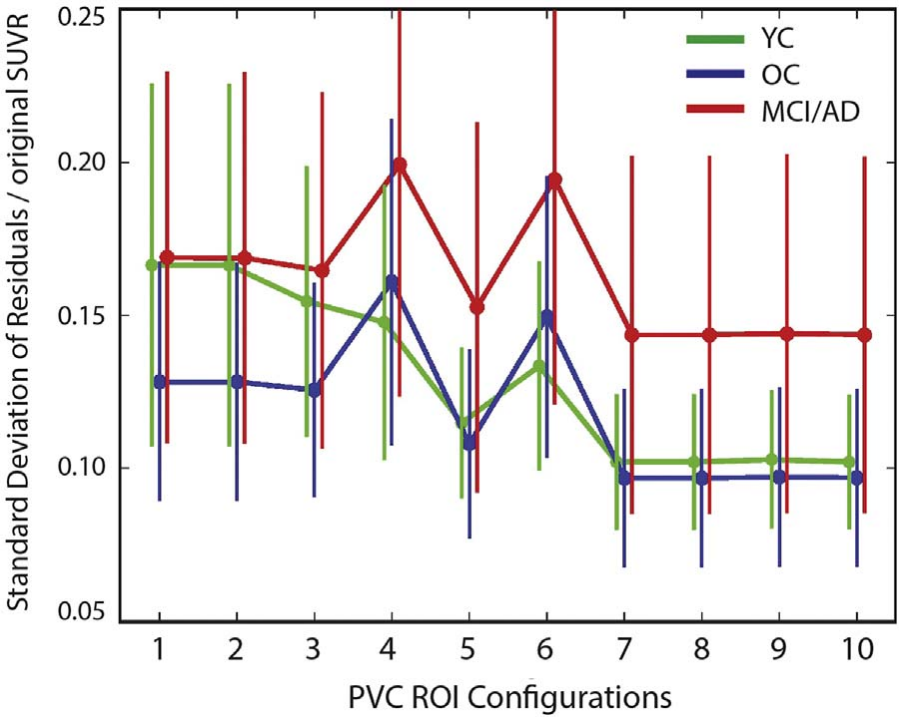
The standard deviation of the residuals normalized by the original SUVR reflects how well the PVC ROI configuration (x-axis) explained the original SUVR data. The lower the value (y-axis), the better the ROI configuration explained the original SUVR data. ROI configuration 1 was all ROIs in Table 1 + choroid plexus. ROI configuration 2, choroid plexus was divided into low and high ROIs. ROI configuration 3 was the same as 2 plus ECH (SUVR threshold=1.6). ROI configuration 4 was the same as 2, no ECHs, and the c3 mask and c4+c5 mask were added. ROI configuration 5 included ECHs (threshold=1.6) and c3 ROI and a c4+c5 ROI. ROI configuration 6 was the same as ROI configuration 4 except the c3 mask and c4+c5 mask were both divided into high and low masks (threshold=1). ROI configuration 7 included ECHs (threshold=1.6), c3 low and high (threshold=1) and c4+c5 low and high (threshold=1) ROIs. ROI configuration 8 was the same as 7 except the inferior cerebellar gray was scanned for high voxels. ROI configuration 9 was the same as 8 except a threshold of 1.3 was used for ECH. ROI configuration 10 was the same as 8 and 9 except a threshold of 1.9 was used for ECH. The ROIs used for calculating the normalized standard deviation corresponded to those in Table 1.

Fig. 2 shows the mean and standard deviation of PVCnstd within each subject group averaged over ROIs 1–77 from Table 2. Including c3 and c4+c5 masks without ECHs in the PVC (steps 4 and 6) causes an increase in the PVCnstd. Therefore, not including ECHs in the ROI configuration results in a worse fit of the data. If steps 4 and 6 are ignored, there was a decrease of PVCnstd from ROI group 2 to 3 to 5 to 7, reflecting an improvement of the fit to the data when ECHs, c3 and c4+c5 masks and splitting those masks into high and low uptake are included. The addition of scanning inferior cerebellar gray for voxels > 1.6 showed no improvement in the model because this rarely occurs. Setting the threshold for defining ECH at 1.3, 1.6, and 1.9 (steps 9, 10 and 8, respectively) also had no effect overall on the success of the PVC from a global quantitative perspective.

ROI configuration 8 was chosen as the final version [6], although we have written the code so that it can flexible. Information about the ROIs added to the ROI configuration for step 8 is found in the description of the code below.

## 3 The code

The main function is TAUPVC_RUNME_Create_ROIs_For_Rousset. The inputs into this Matlab function are full paths and filenames for: 1. aparc+aseg.nii from FreeSurfer, 2. c1.nii, c2.nii, c3.nii, c4.nii, and c5.nii from SPM12, 3. Reverse normalized Cerebellar SUIT template (resliced to the dimensions of the MRI), 4. Mean or sum of realigned [^18^F]-AV-1451 frames coregistered and resliced to the MRI, and 5. Approximate PET scanner resolution. The outputs are 1. [^18^F]-AV-1451 normalized to inferior cerebellar gray, and 2. an edited version of aparc+aseg file in which voxels from each ROI used for PVC are assigned a different integer value, 3. an SUVR image normalized by the inferior cerebellar gray cortex with no partial volume correction, and 4. PV-corrected values are saved in variable roigroups in a final FINAL0_roigroups.mat.

The first step in the code was to assign new index values and group ROIs (as described in Table 2) using the aparc+aseg.nii file from the FreeSurfer segmentaion. Ventricles were unassigned and therefore assumed to have a 0 PVC value.

**Fig. 3:**
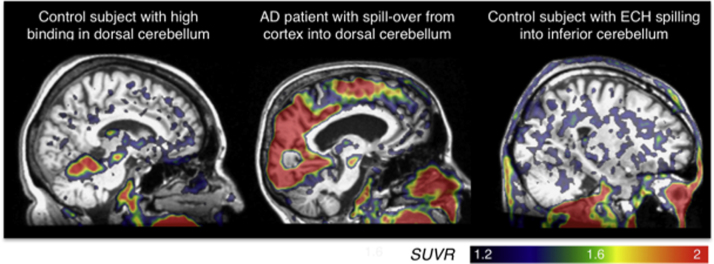
Examples of binding in dorsal cerebellum, bleed in from neighboring ROIs into dorsal cerebellum, as well as bleed in from ECH into inferior cerebellum.

The second step was to create the inferior cerebellar gray ROI from the reverse-normalized cerebellar SUIT template (rnCereSUIT). The inferior cerebellar gray was used as the reference region due to frequent binding seen in the dorsal cerebellum as well as bleeding in from adjacent cortical regions (Fig. 3). The rnCereSUIT was divided into binary masks for the inferior portion (indices 6, 8–28, 33 and 34) and the superior portion (1–5 and 7) of the cerebellar gray. These masks were smoothed by 8mm^3^, ensuring that all voxels defined as cerebellar gray in the original aparc+aseg.nii had a non-zero value in either the smoothed inferior or smoothed superior mask. This was necessary because the original aparc+aseg cerebellar gray region did not perfectly overlap with rnCereSUIT. If a voxel was defined as cerebellar gray in the original aparc+aseg.nii and the smoothed inferior mask > smoothed superior mask, it was added to the final inferior cerebellar gray ROI. If a voxel was defined as aparc+aseg cerebellar gray and the smoothed superior mask > smoothed inferior mask, it was added to the final superior cerebellar gray ROI. A new SUVR was calculated by normalizing the [^18^F]-AV-1451 SUVR by the mean inferior cerebellar gray; this newly-normalized SUVR was used for the rest of the code.

**Fig. 4:**
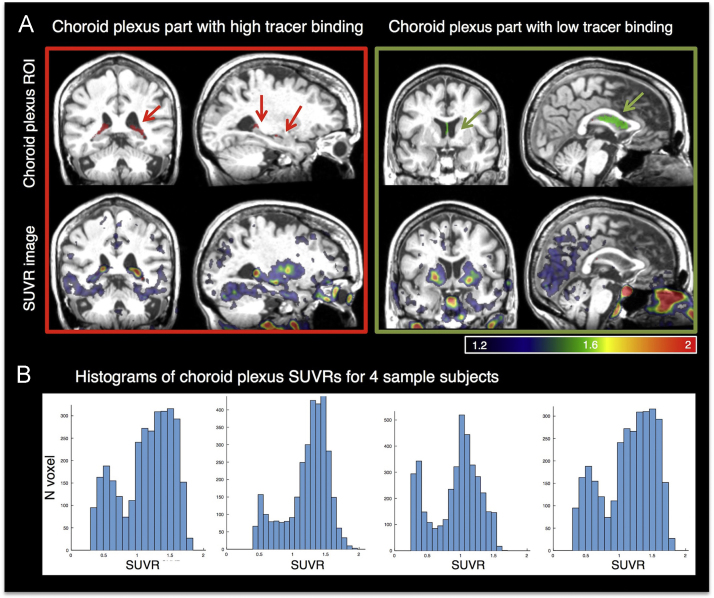
Areas of high (A) and low (B) choroid plexus binding are shown in the masks (top row) and SUVR image (bottom row) for one sample subject. Histograms from 4 subjects show the bimodal distribution of the choroid plexus [^18^F]-AV-1451 binding.

**Supplementary Fig. 1:**
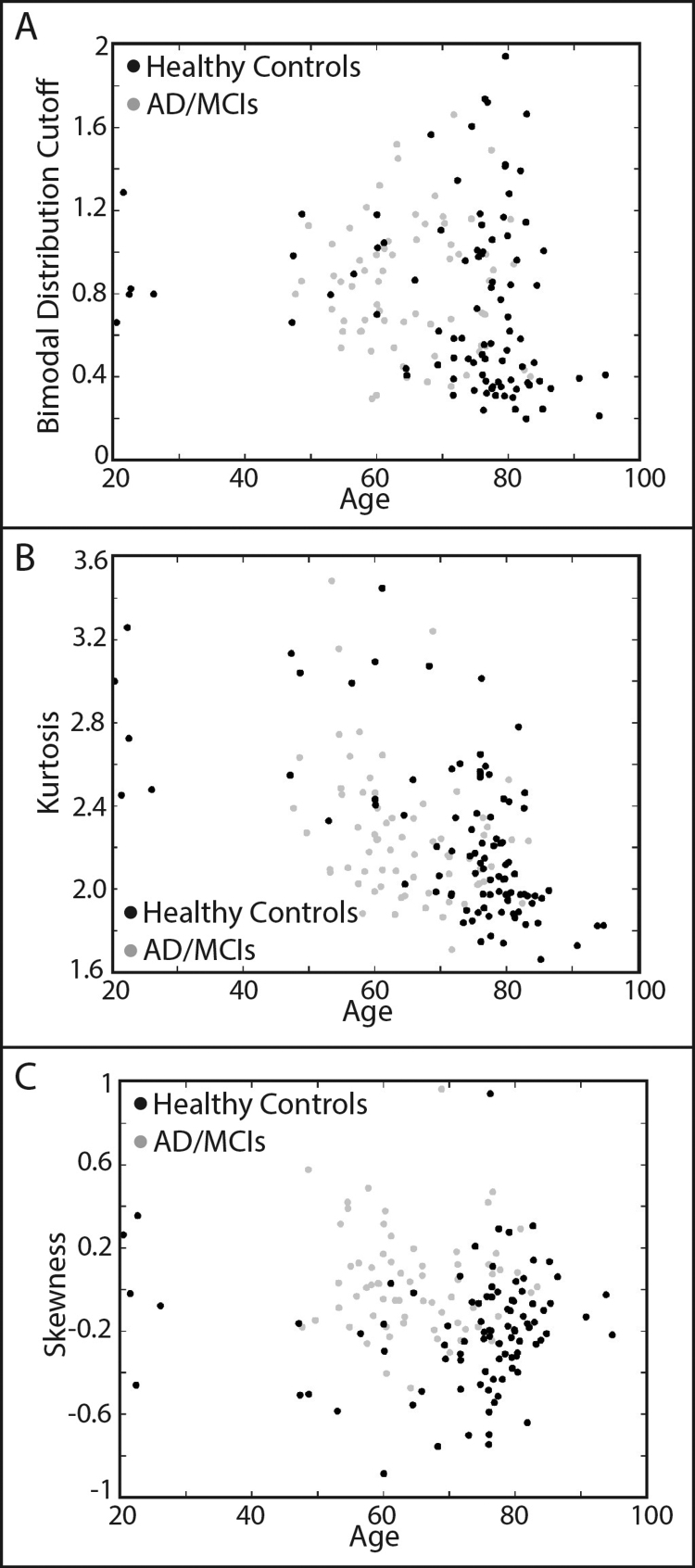
For each subject, the whole choroid plexus distribution of SUVR values was analyzed. A. shows the cutoff for each subject of the bimodal distribution, B. the kurtosis and C. the skewness.

The third step was to take the choroid plexus, as segmented by FreeSurfer, and divide it into low and high choroid plexus ROIs (Fig. 4). The choroid plexus was not a homogenous ROI but showed a bimodal distribution of tracer binding and therefore had to be divided into choroid plexus high ROI and low ROI. The ventral portion of the choroid plexus that is next to the hippocampus shows off-target binding (Fig. 4A, red arrows). In order to realistically quantify the activity in the hippocampus, this had to be addressed with PVC. Bimodal distribution cutoff, kurtosis and skewness is shown in Supplementary figure 1 for the whole choroid distribution across all subjects. We defined high and low choroid plexus ROIs based on if voxels had more or less binding than the reference region. Within the choroid plexus, the low choroid plexus ROI contained voxels where the SUVR<=1 and had at least 100 contiguous voxels; the high choroid plexus ROI contained voxels where SUVR>1 and had at least 100 contiguous voxels. This did not account of all choroid plexus voxels. So the high and low choroid plexus masks were smoothed (8 mm^3^). Any unassigned voxels within aparc+aseg choroid plexus were assigned to high choroid plexus if smoothed high choroid plexus > smoothed low choroid plexus, and low choroid plexus if smoothed low choroid plexus > smoothed high choroid plexus. In the edited_aparc+aseg.nii, high choroid plexus voxels=79 and low choroid plexus voxels=80.

**Fig. 5:**
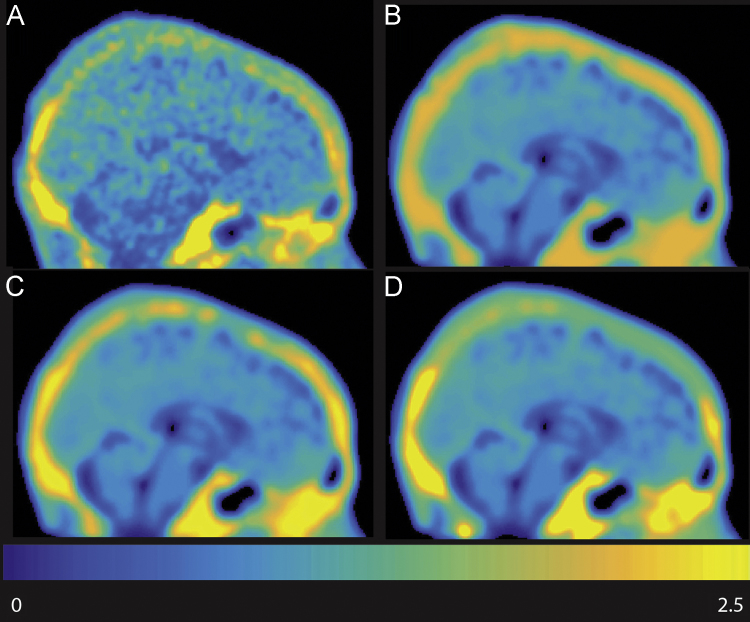
young healthy control subject with ECH near the occipital lobe. A is the original SUVR image. B has the calculated pre-PVC for the ROI group with an ECH threshold of 1.3. C is the same as B but ECH threshold was 1.6, and D had an ECH threshold of 1.9.

The fourth step was to define ECHs. ECHs are defined in each subject's native space, and are defined on an individual basis. This was done in order to make no assumptions regarding the location and spread of ECHs. As mentioned above, we explored 3 possible thresholds (1.3, 1.6, 1.9) for ECH before finalizing our PVC code. They resulted in similar values for PVCnstd for cortical ROIs, so we relied on qualitative inspection of the calculated pre-PVC images in comparison to the original SUVR. Fig. 5 is an example of such an image. A threshold of 1.3 included too many voxels and 1.9 at times resulted in too small ECHs, 1.6 seemed to be the best compromise.

**Fig. 6:**
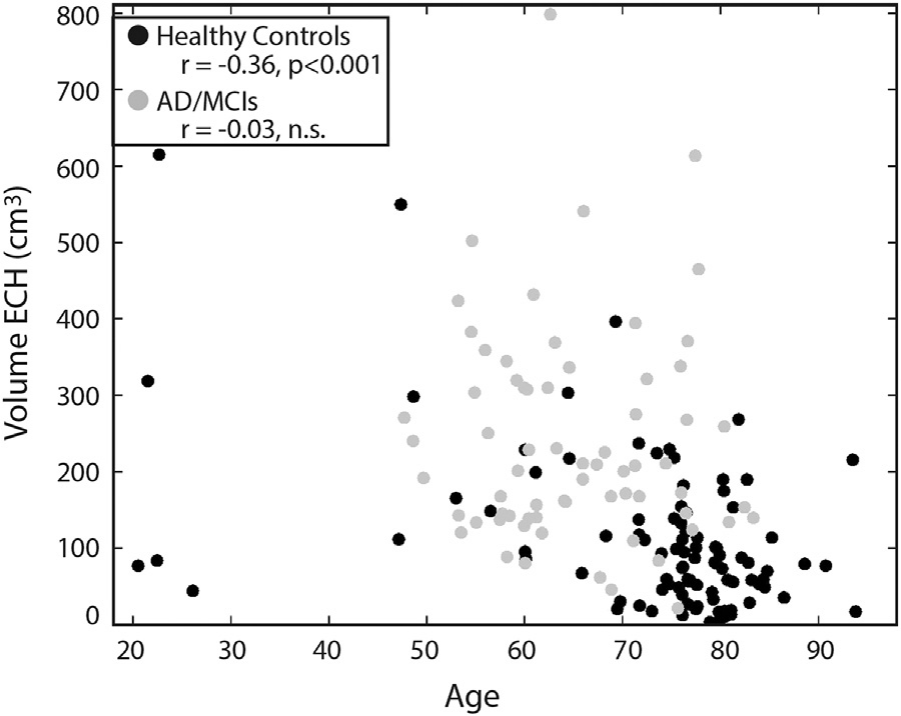
Age versus volume of ECH; Spearman correlation shows age is correlated with the volume of ECH in healthy controls but is not significant in the AD/MCI subgroup within that subsample.

In the implementation, we wanted to look for ECHs in c4 and c5 (skull+meninges), but also some of c3 that was not close to cortex. c3 is defined as CSF in SPM12 but this probability mask also contains parts of dura and soft tissue where ECHs have occurred, unrelated to cortical activity. To define the ECH-searchable ROI, we used the SPM12 segmentation c3, c4, and c5 probability maps, a brain mask, and the SUVR. The brain mask was a binary mask of the non-zero voxels in the aparc+aseg, this smoothed to the resolution of the scanner. The c4 and c5 probability maps were added together, a binary mask of any voxels in c4+c5 > 0.5 was created and smoothed to the resolution of the scanner. We then searched in the c3 probability mask: any voxels with a probability >0.5 of being c3 (CSF) and that were closer to the c4+c5 mask than to the brain mask (smoothed c4+c5 mask > smoothed brainmask) were added to the ECH-searchable ROI. Any voxels where c4+c5>0.3 were also added to the ECH-searchable ROI. Within the ECH-searchable ROI, voxels with SUVR>1.6 were considered probable ECHs. Contiguous probable ECH voxels were separated into individual clusters, and considered a full-fledged ECH if the cluster contained > 500 voxels. Each ECH was added to the edited_aparc+aseg with a unique integer value starting at 85 and counting up for each subsequent ECH. We found that the number of overall ECH voxels was negatively correlated to age in healthy controls (p<0.001, Fig. 6). However, the correlation with age to number of ECH voxels was not significant when looking only in healthy female controls (n=51, age range = 53–93.5 years) but was significant (p<0.001) in age-matched healthy male controls (n=34, age range = 56.5–93.8).

The fifth step was to assign remaining c1, c3 and c4+c5 voxels that were not defined in the original aparc+aseg.nii (aparc+aseg=0) and were not classified as ECHs. Voxels were defined as CSF low (or c3 low) if 1. CSF (c3) or gray matter (c1) probability >0.3, 2. CSF (c3) probability > 0, 3. CSF (c3) probability > bone + meninges probability (c4+c5) and 4. SUVR was > 0.1 and < 1. CSF high met the same criteria except SUVR was >=1. For bone + meninges low (c4+c5 low), c4+c5 had to have a probability > 0.3, c4+c5>c3 and SUVR > 0.1 and < 1. Bone + meninges high met the same criteria as bone + meninges low except SUVR was >=1. In the edited_aparc+aseg file, CSF low was assigned a value of 81, CSF high=82, bone + meninges low = 83, and bone + meninges high = 84. The SPM12 gray matter mask (c1) was included in the search because we found there was not 100% overlap between FreeSurfer defined gray matter voxels and SPM12 defined gray matter voxels. In this case, if a voxel was not defined as gray matter in FreeSurfer, but SPM12's probability of the voxel being gray matter was 30% or higher, we assumed SPM12 misclassified the voxel and it should be classified as CSF.

Next, voxels with gray matter mask (c1) > 0.3, 0% probability of being c3, c4, or c5, SUVR>0.1, and unassigned in the edited_aparc+aseg (=0) were addressed. For each of these voxels, a matlab function looked at the integer value of the surrounding voxels and assigned the stray voxel the integer value most common to its neighbors. We wanted to assign unassigned voxels because most of these voxels were gray matter missed by the FreeSurfer segmentation that showed some tracer uptake, and if left unassigned the value would assume to be 0 during PVC.

Lastly, the inferior portion of the cerebellar gray was searched for clusters of voxels with SUVR>1.6 and more than 500 contiguous voxels in a cluster (Fig. 3). If any existed, each was individually assigned a unique integer value (counting up after the highest ECH integer value) in the edited_aparc+aseg. A new mean of the inferior cerebellar gray was calculated with these nuisance reference region hotspots removed, the SUVR was normalized one last time to this new mean of the inferior cerebellar gray. Across all subjects in [6] (n=216) inferior cerebellar ECH was only found in three subjects.
