# Supplementary material for: Considerations and code for partial volume correcting [18F]-AV-1451 tau PET data
Source: Data Brief. 2017 Oct 16;15:648–57. doi: 10.1016/j.dib.2017.10.024 (PMC5671473; doi:10.1016/j.dib.2017.10.024)
Supplement: Supplementary file 1 — Supplementary material [file mmc1.docx]

Conflict of Interest:

Suzanne Baker and Anne Maass have nothing to disclose. Dr Jagust has served as a consultant to Genentech, Novartis, Biogen, and Bioclinica
